# Effective doping control in Sm-doped BiFeO_3_ thin films *via* deposition temperature†

**DOI:** 10.1039/d0ra06775j

**Published:** 2020-11-04

**Authors:** Han Wang, Jijie Huang, Xing Sun, Jie Jian, Juncheng Liu, Haiyan Wang

**Affiliations:** School of Materials Engineering, Purdue University West Lafayette IN 47907 USA han.wang@pnnl.gov hwang00@purdue.edu; School of Electrical and Computer Engineering, Purdue University West Lafayette IN 47907 USA

## Abstract

Sm-doped BiFeO_3_ (Bi_0.85_Sm_0.15_FeO_3_, or BSFO) thin films were fabricated on (001) SrTiO_3_(STO) substrates by pulsed laser deposition (PLD) over a range of deposition temperatures (600 °C, 640 °C and 670 °C). Detailed analysis of their microstructure *via* X-ray diffraction (XRD) and transmission electron microscopy (TEM) shows the deposition temperature dependence of ferroelectric (FE) and antiferroelectric (AFE) phase formation in BSFO. The Sm dopants are clearly detected by high-resolution scanning transmission electron microscopy (HR-STEM) and prove effective in controlling the ferroelectric properties of BSFO. The BSFO (*T*_dep_ = 670 °C) presents larger remnant polarization (Pr) than the other two BSFO (*T*_dep_ = 600 °C, 640 °C) and pure BiFeO_3_ (BFO) thin films. This study paves a simple way for enhancing the ferroelectric properties of BSFO *via* deposition temperature and further promoting BFO practical applications.

## Abstract 2

Sm-doped BiFeO_3_ (Bi_0_._85_Sm_0.15_FeO_3_) thin films were fabricated on (001) SrTiO_3_ substrates by PLD over a range of deposition temperatures. The Sm dopants are clearly detected by high-resolution scanning transmission electron microscopy.

## Graphical abstract

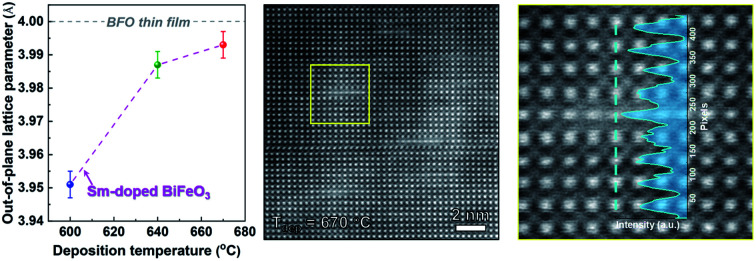

As a promising alternative lead-free piezoelectric material, BiFeO_3_ (BFO) thin films have attracted enormous research interest due to their remarkable multiferroic properties and piezoelectric response.^1–4^ However, the high leakage current and large coercive field are factors limiting the extensive use of BFO.^5^ Since the polarization is mainly induced by the Bi^3+^ ions, various rare-earth elements (*e.g.*, La, Nd, Sm, and Gd) have been doped into the Bi-site to improve the overall ferroelectric response.^6–8^ According to the site-engineering concept, the doping of foreign elements causes chemical pressure and controls the volatility of Bi atoms in BFO systems.^9,10^ The structural information of rare-earth-doped BFO (Re-BFO) systems is therefore upgraded and a preliminary phase diagram is proposed.^11–14^

Sm-doped BFO (Bi_1−*x*_Sm_*x*_FeO_3_, BSFO) thin film has attracted research attention, due to the narrow concentration range at room temperature.^15^ It shows ferroelectric (FE) rhombohedral to antiferroelectric (AFE) orthorhombic phase transitions as the Sm doping amount increases. Morphotropic phase boundary (MPB) appears at around *x* = 0.13–0.15, and high values of out-of-plane piezoelectric coefficient (*d*_33_ ∼ 110 pm V^−1^) and enhanced dielectric constant at *x* = 0.14 are reported in such systems.^16,17^ Structure studies show that three main phase, FE *R*3*c* phase, AFE PbZrO_3_-like phase, and paraelectric *Pnma* phase, coexist at the MPB composition.^18^ In this regard, many efforts have been made on the investigation of phase transitions under external stimuli.^19,20^ However, there are not much work on the structure analysis of BSFO at the atomic-scale level. It will be interesting to investigate the microstructure with high-resolution transmission electron microscopy (TEM) and high-resolution scanning transmission electron microscopy (HR-STEM). The detailed microstructure information will reveal how the Sm dopants distribute in the overall BSFO lattice. In addition, BSFO films in previous studies are mostly prepared by solid phase synthesis and sol–gel method^15,20,21^ with very few reports using pulsed laser deposition (PLD).^17,22^ In those prior reports, Sm-doping amounts in films were controlled by changing the composition of the deposition targets. Deposition temperature has been proven as an effective parameter for PLD in controlling the doping amount, thin film microstructure and the related properties. In the Ag-doped ZnO (SZO) system, deposition temperature was directly used to control the density of stacking faults and consequently affect the electrical transport properties.^23^ Table S1† lists several reported deposition temperatures for Re-BFO thin films, which is in the range of 520 °C to 850 °C. Here, we used three different substrate temperatures, 600 °C, 640 °C and 670 °C, for the BSFO film fabrication *via* PLD. Pure BFO film was also grown as a reference sample for comparison. Besides the detailed microstructure, the corresponding ferroelectric property measurements were conducted on the BSFO thin films to investigate how the Sm dopants affect the ferroelectric behavior of BSFO.

In the present work, Bi_0.85_Sm_0.15_FeO_3_ (BSFO) was selected as the model system. The BSFO target was synthesized by a conventional solid-state sintering method using high-purity Bi_2_O_3_ (99.99%), Fe_2_O_3_ (99.95%) and Sm_2_O_3_ (99.90%) powders. Thin films were grown on (001) single-crystal SrTiO_3_ (STO) substrates epitaxially *via* PLD. KrF excimer laser with a wavelength of 248 nm was used as the laser source. Three different substrate temperatures, 600 °C, 640 °C and 670 °C, were applied in the deposition. For all depositions, oxygen partial pressure was kept at 200 mTorr and the deposition rate was 5 Hz. The films were cooled down to room temperature at a cooling rate of 10 °C min^−1^ in 200 torr oxygen atmospheres. The growth condition and parameters of BFO film is same with our previous report.^24^ Au top contacts with 100 nm thickness and 0.1 mm^2^ were deposited by a custom-built magnetron sputtering system. The Au sputter target (99.99% pure) is made by Williams Advanced Materials.

X-ray diffraction (XRD) spectra were collected by a PANalytical Empyrean system using Cu K_α_ radiation. The Raman spectra were measured by Renishaw's inVia Raman microscope. The microstructure analysis was performed on FEI TALOS F200X TEM/STEM operated at 200 kV. The energy-dispersive X-ray spectroscopy (EDS) chemical mapping was acquired by the SuperX EDS system with four silicon drift detectors. Ferroelectric characterization was conducted by Precision LC II Ferroelectric Tester (Radiant Technologies, Inc.).

Fig. 1(a) shows the *θ*–2*θ* XRD spectra of the as-prepared thin film samples deposited at 600 °C, 640 °C and 670 °C. All the films display BSFO (00*l*) diffraction peaks, indicating highly textured BSFO along *c*-axis. It is noted that BSFO (003) peak shifts from 71.62°, to 70.82° and to 70.70° with the deposition temperature increasing from 600 °C to 640 °C, and 670 °C. The corresponding out-of-plane lattice parameters are calculated to be 3.951 Å, 3.987 Å and 3.993 Å, respectively and are summarized in Fig. 1(b). Compared with the out-of-plane lattice parameter of pure BFO film (∼4.000 Å) on STO substrate,^24^ three BSFO samples show smaller lattice parameters than that of pure BFO. This is because partial Bi^3+^ (radius = 1.030 Å) ions have been substituted by Sm^3+^ ions with smaller radius (radius = 0.958 Å).^25^ The BSFO peak at around 32.155° (denoted as “*”) in Fig. 1(a) comes from rhombohedral (110) peak and the peak disappears when the deposition temperature increases to 670 °C. The above results indicate that the deposition temperature influences the Sm-doping amount and the BSFO crystal structure. Raman analysis has been conducted on all BSFO and pure BFO films. The Raman spectra were fitted with Lorentzian curves, as shown in Fig. S1.† The reported data of bulk polycrystalline Bi_1−*x*_Sm_*x*_FeO_3_ was taken as reference.^26^ The overall shape of peaks is the same, which implies that the main structure of BSFO sample is the same as the rhombohedral *R*3*c* structure of BFO.

**Fig. 1:**
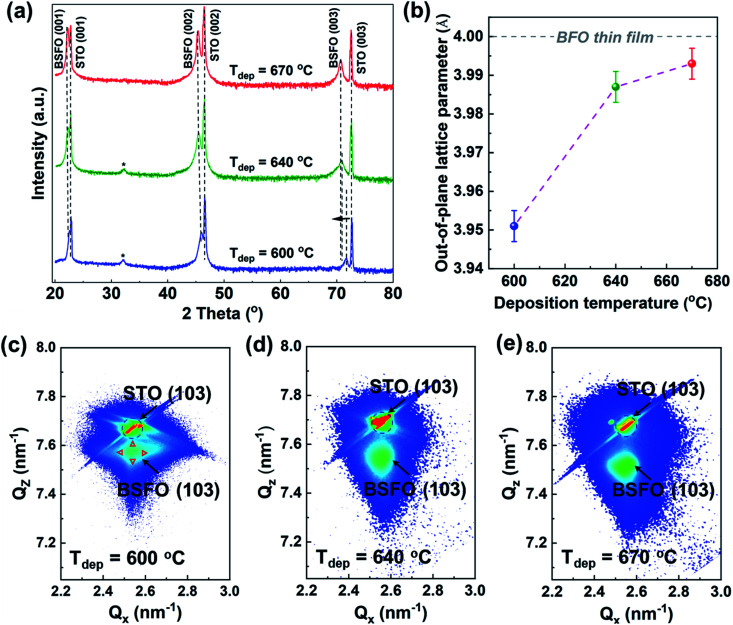
(a) *θ*–2*θ* XRD spectra of BSFO film deposited at 600 °C, 640 °C and 670 °C. (b) The summary of out-of-plane lattice parameter. (c–e) Reciprocal space map (RSM) results of BSFO (103) peaks.

To further analyze the detailed phase information, asymmetric reciprocal space mapping (RSM) measurements were performed around the (103) diffraction peak for all the three BSFO samples and the results are shown in Fig. 1(c)–(e). The RSM pattern of sample (*T*_dep_ = 600 °C) exhibits four domains, which are shown by red triangles. It indicates the existence of rhombohedral-like phase which has antipolar nature.^27–29^ For the other two samples (*T*_dep_ = 670 °C and *T*_dep_ = 640 °C), peak split along *Q*_*x*_ direction is not apparent and the narrower width along *Q*_*x*_ direction is observed, indicating less structure distortion in BSFO films deposited at higher temperature. These results provide direct evidence that the deposition temperature significantly affects the domain structure of the BSFO film.

In order to analyze the microstructure structure, TEM analysis has been applied on two samples (*T*_dep_ = 670 °C and *T*_dep_ = 600 °C). Fig. 2(a) and (c) show the overall films stacks of BSFO on STO substrates. The corresponding selected area electron diffraction (SAED) patterns of BSFO thin films only demonstrate the rhombohedral-like phases. It is interesting to note that the TEM image of high deposition temperature sample (*T*_dep_ = 670 °C) exhibits few dark lines. And the dark line density in the high deposition temperature sample is obviously higher than that in the lower deposition temperature one. The image contrast is proportional to ∼*Z*^2^ (*Z*, atomic number) in TEM bright-field mode. The dark line is therefore proposed be related with Sm, owing to *Z*_Sm_ is larger than *Z*_Bi_ and *Z*_Fe_. It also suggests that the higher deposition temperature introduce more Sm dopants in BSFO films than the lower one. The Fig. 2(b) and (d) are high resolution TEM (HR-TEM) images from local areas, which were analyzed by Fast Fourier Transform (FFT). The spots (marked by red arrows) in the FFT image of the sample (*T*_dep_ = 670 °C) correspond to the incommensurate phase, which is caused by the competition between ferroelectric (FE) and antiferroelectric (AFE) phases. Different phase emerges in the sample (*T*_dep_ = 600 °C), and the corresponding spots are marked by red circle. It has been proved as the antipolar orthorhombic AFE phase, linked to the macroscopic AFE behavior. The phase information of Bi_0.85_Sm_0.15_FeO_3_ thin film in this study is different from previous reported BSFO with only AFE phases.^16^ The above microstructure analysis shows the effect of deposition temperature on the phase formation in BSFO system even with 15 atomic percent Sm.

**Fig. 2:**
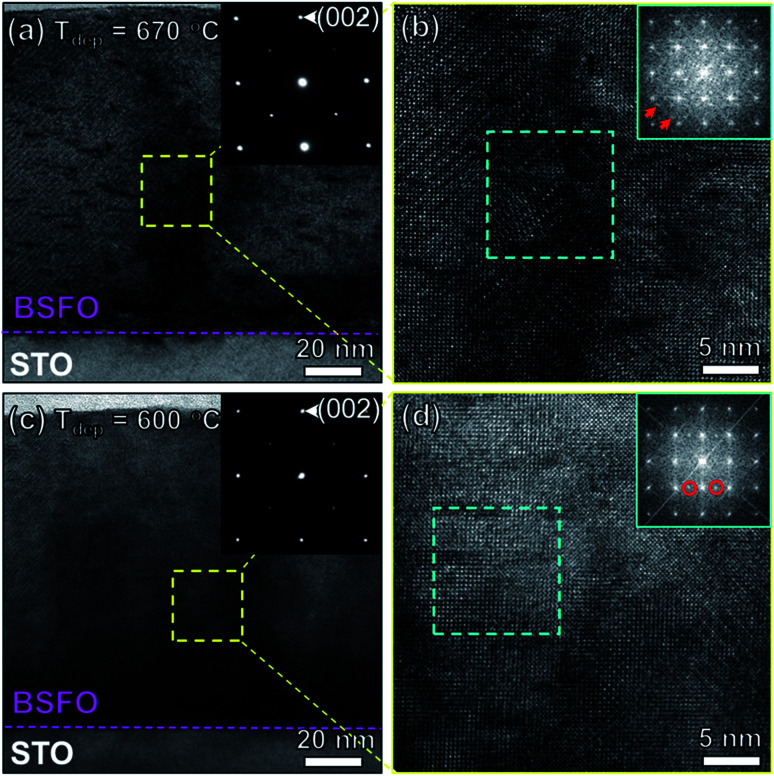
(a and c) Cross-sectional TEM images of BSFO thin films (670 °C and 600 °C) on STO substrates. (b and d) High resolution TEM images. The insets in (a and c) show the corresponding (SAED) pattern of BSFO thin films. The insets in (b and d) show the fast-Fourier transformed (FFT) images from the blue squared region.

STEM analysis was then performed to resolve the composition information. Fig. 3(a) shows the high-resolution STEM (HR-STEM) image of the 670 °C sample. The high angle annular dark field mode (HADDF) image intensity is proportional to the atomic number *Z*. Thus, the white line areas in STEM image correspond to the dark lines in TEM images. We further examined the composition distribution across this Sm layer using the intensity line profile (Fig. 3(b)), which provides a direct interpretation of composition information in the HAADF imaging mode. It is obvious that the position near white line area has higher intensity than other areas. This result proves that Sm^3+^ ion has been doped into BFO system effectively. Energy dispersive spectroscopy (EDS) measurements have been further applied on the same area. The EDS mapping of Bi and Sm elements are shown in Fig. S2(b) and (c).† The EDS line-scan analysis of Sm is overlaid on the HADDF image and displayed in Fig. S2(a).† The line profile reflects high content of Sm corresponding to the white line area. Therefore, both the TEM and STEM results show that the higher deposition temperature BSFO sample (*T*_dep_ = 670 °C) has higher Sm-doping amount.

**Fig. 3:**
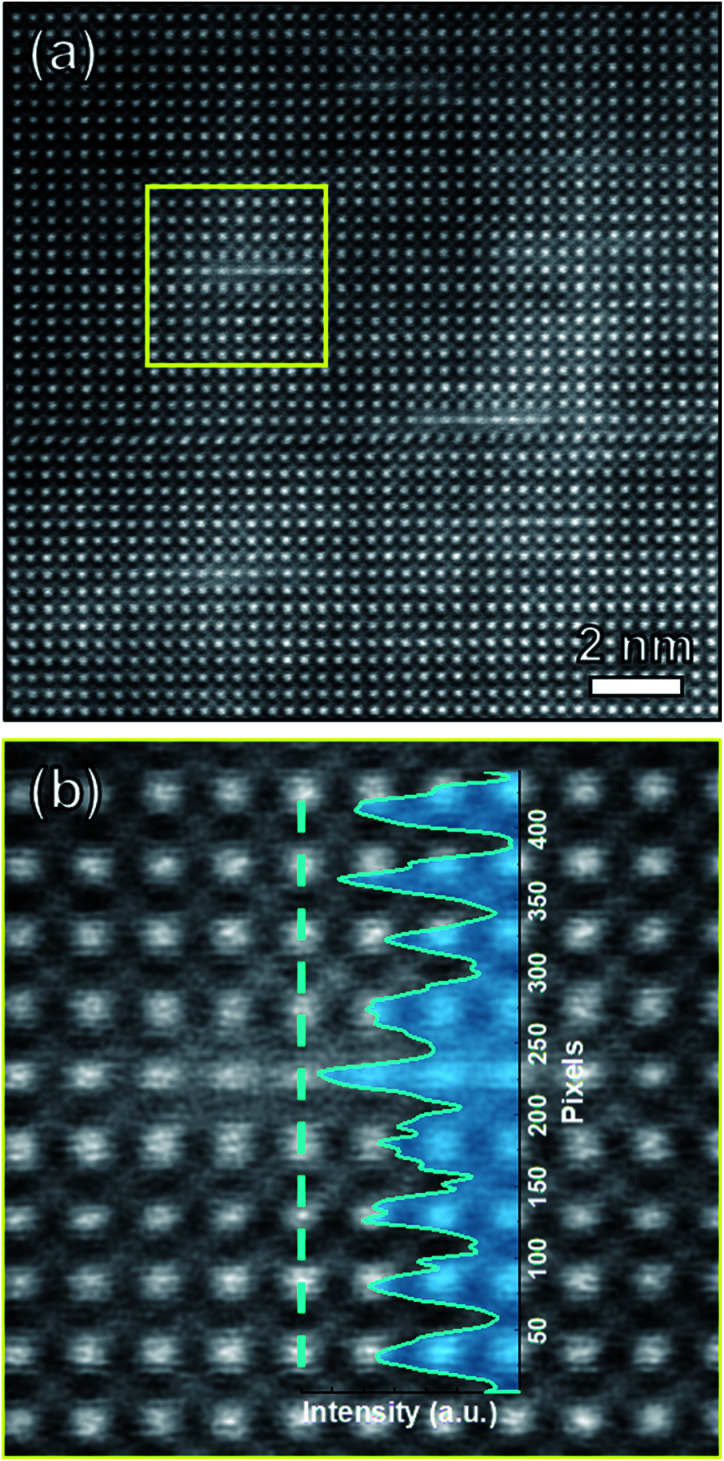
(a) HR-STEM image of BSFO film deposited at 670 °C. (b) Enlarged view of the yellow dashed square area from (a). The intensity line profile is inserted along the marked blue line.

The ferroelectric behaviors were characterized by the polarization–electric field (*P*–*E*) hysteresis loops. Fig. 4(a)–(c) show the *P*–*E* loops of BSFO samples with three different deposition temperatures, while Fig. 4(d) shows the loop of pure BFO as a comparison. The polarization measurement as a function of electric field measurement was carried out at room temperature for several times to ensure the reproducibility of the measurements. The BSFO sample (*T*_dep_ = 670 °C) with higher Sm-doping amount exhibits obvious enhanced polarization. The remnant polarization (Pr) for the film is determined to be 17 μC cm^−2^, much larger than other two BSFO samples and the BFO sample. It was proposed that the incorporation of Sm could break the short-range dipolar regions, surmount the local barrier and transform it to the long-range polar structure.^30^ The BFO film with four-variant domains exhibits a lower electric filed and remnant polarization Pr.^31^ It was also found that the formation of bridging phase could enhance piezoelectric and dielectric properties of BSFO.^16^ In this work, the BSFO sample (*T*_dep_ = 600 °C) shows four structural domain structure and the lowest Pr. With the increase amount of Sm-doping amount, incommensurate phase appears in the 670 °C sample. We conclude that the higher deposition temperature introduces the higher Sm-doping amount, which further assists the incommensurate phase formation and suppresses the AFE phase. In addition, the unsaturated *P*–*E* loop of BFO film indicates it suffers from high leakage current, which is generally due to the appearance of Bi deficiencies and oxygen vacancies.^32^ The Sm–O bond enthalpy (565 ± 13 kJ mol^−1^) is stronger than the Bi–O bond enthalpy (337 ± 12.6 kJ mol^−1^).^33^ Therefore, the higher polarization exhibited by BSFO samples (*T*_dep_ = 670 °C) than the pure BFO proves that the Sm dopant could compensate for the Bi loss and suppress the formation of oxygen vacancies. The *P*–*E* loop results were compared with prior reports on Re-BFO films. As shown in Table S1,† the remnant polarization is quite different for Re-BFO systems. It is due to the different ionic radii of the rare earth elements, which result in different structural distortion of BFO and diverse critical doping ratio for phase transitions. Besides the phase variants, the polarization is closely related with the orientation of crystalline structures.

**Fig. 4:**
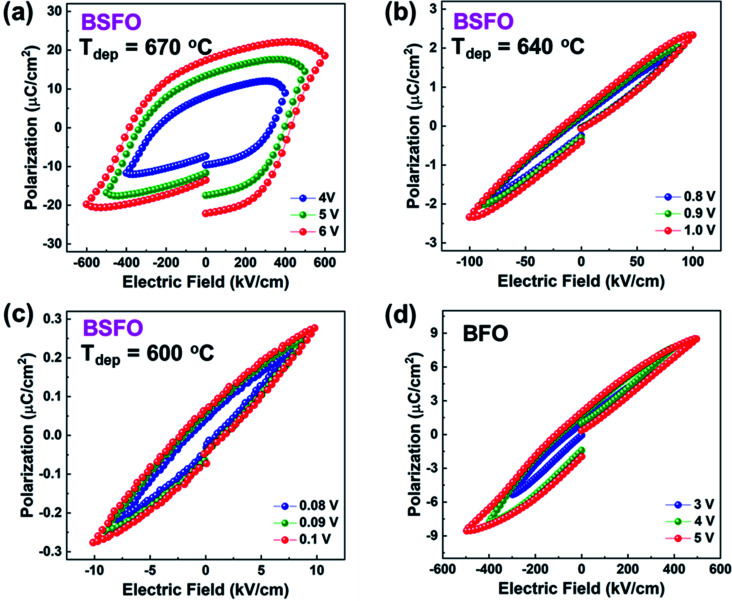
(a–c) Polarization hysteresis measurements for BSFO film deposited at 670 °C, 640 °C and 600 °C. (d) Polarization hysteresis measurements for BFO film.

This study demonstrates that Sm-doping amount in BSFO thin films can be effectively tuned *via* deposition temperature. The Sm dopants influence phase formation of BSFO and further control the macroscopic ferroelectric properties. The local incommensurate phase presented by Bi_0.85_Sm_0.15_FeO_3_ with higher Sm-doping amount than the reported ones (Bi_0.86_Sm_0.14_FeO_3_), which is extremely helpful in constructing phase diagram of BSFO. More interestingly, the existence and location of Sm dopants in BSFO thin film have been directly demonstrated by the HR-STEM and corresponding EDS analysis. This work is also beneficial for the exploration of other Re-BFO films with deposition temperatures and detailed structure analysis, which is an important step toward the practical applications of Re-BFO in electronic devices.

## Data availability

The data that support the findings of this study are available from the corresponding author upon reasonable request.

## Conflicts of interest

There are no conflicts to declare.

## Supplementary Material

RA-010-D0RA06775J-s001
