# Supplementary material for: Effective doping control in Sm-doped BiFeO3 thin films via deposition temperature
Source: RSC Adv. 2020 Nov 4;10(66):40229–33. doi: 10.1039/d0ra06775j (PMC9057518; doi:10.1039/d0ra06775j)
Supplement: RA-010-D0RA06775J-s001 [file RA-010-D0RA06775J-s001.pdf]

Electronic Supplementary Material (ESI) for RSC Advances.
This journal is © The Royal Society of Chemistry 2020

Supplementary Information

Effective doping control in Sm-doped $BiFeO_3$ thin films via deposition temperature

Han Wang[1*], Jijie Huang[1], Xing Sun[1], Jie Jian[1], Juncheng Liu[1] and Haiyan Wang[1,2*]

[1]School of Materials Engineering, Purdue University, West Lafayette, IN 47907, USA.

[2]School of Electrical and Computer Engineering, Purdue University, West Lafayette, IN 47907, USA.

[*]corresponding author: Han Wang: han.wang@pnnl.gov

Haiyan Wang: hwang00@purdue.edu

Table S1 Comparison of rear-earth doped BFO and BFO thin films prepared by PLD

System	Deposition temperature(°C)	Remnant Polarization(μC/cm²)	References
$Bi_{0.8–0.85}Sm_{0.2–0.15}FeO_3$	600	60	1(17)
$Bi_{1−x}RE_xFeO_3$ (RE = Sm, Gd, Dy)	590	15 ~ 70	2(22)
$Bi_{1−x}Nd_xFeO_3$ (x=0.00, 0.05, 0.10 and 0.15)	600	----	3
$Bi_{1−x}Eu_xFeO_3$ (x = 0, 0.03, 0.05, 0.07, 0.1)	700	----	4, 5(30)
$Bi_{0.9}Gd_{0.1}FeO_3$	550	40	6
Tb-doped $BiFeO_3$ ($Bi_{1−x}Tb_xFeO_3$, $0 \le x \le 0.15$)	520	25 ~ 80	7
Yb-doped $BiFeO_3$ ($Bi_{0.85}Yb_{0.15}FeO_3$, YBFO)	850	41.5	8
$BiFeO_3$	650 ~ 700	50	9(26)
$BiFeO_3$	670	----	10(27)
$Bi_{0.85}Sm_{0.15}FeO_3$	670	17	This work

The number in brackets is the sequence in manuscript.

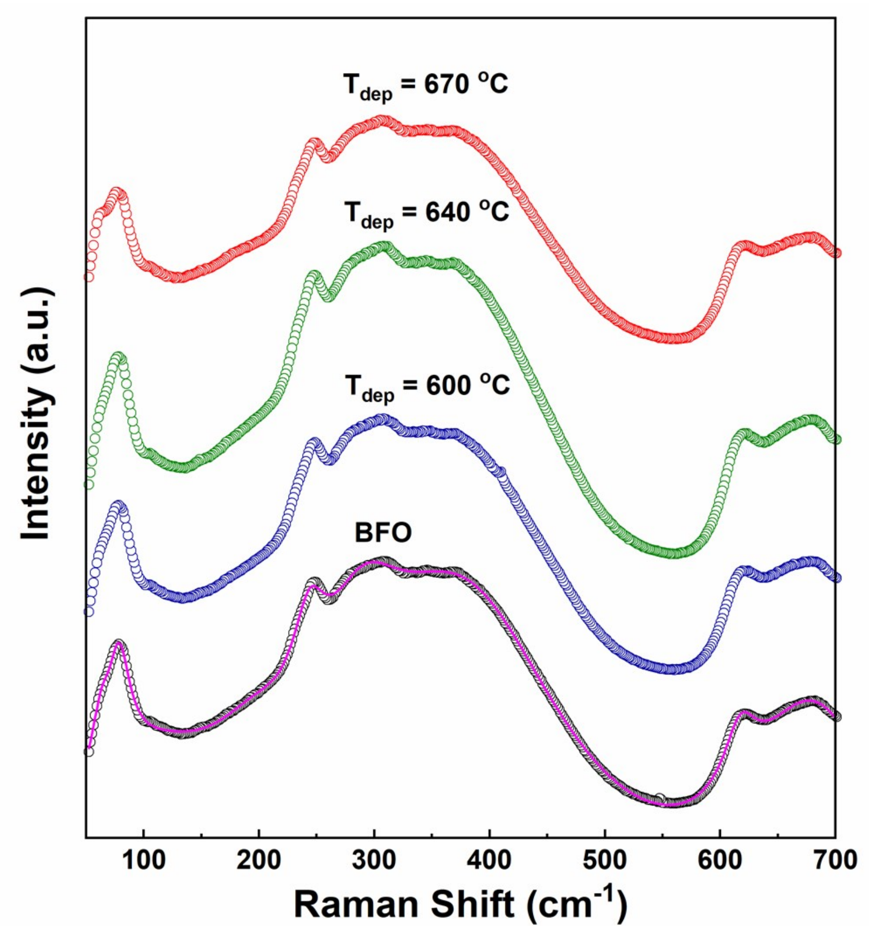

Fig. S1. Raman spectra for BSFO and BFO films.

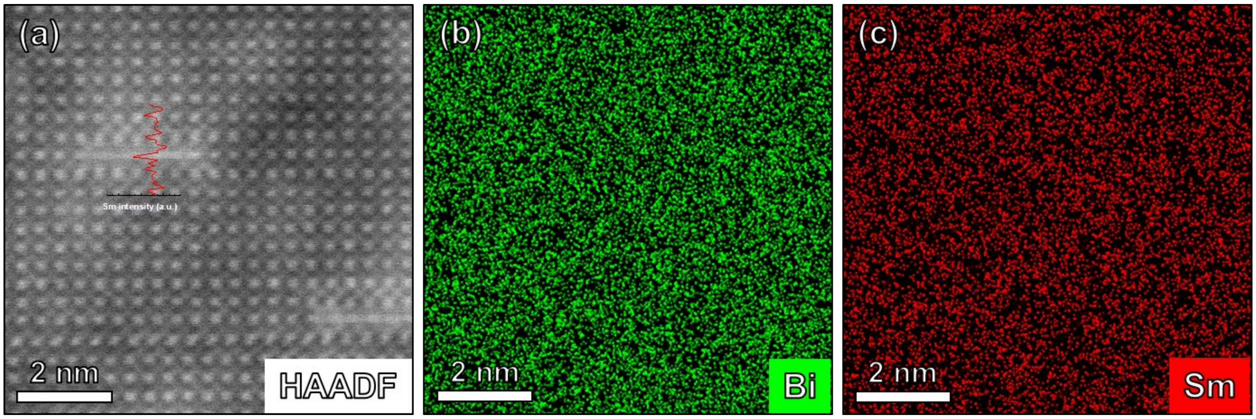

Fig. S2. HAADF image (a) with EDS mapping of Bi (b) and Sm (c) for BSFO thin film. The overlays in (a) shows the EDS line-scan profiles of Sm across the white line.

References:

1. S. Fujino, M. Murakami, V. Anbusathaiah, S.-H. Lim, V. Nagarajan, C. Fennie, M. Wuttig, L. Salamanca-Riba and I. Takeuchi, *Applied Physics Letters*, 2008, **92**, 202904.
2. D. Kan, L. Pálová, V. Anbusathaiah, C. J. Cheng, S. Fujino, V. Nagarajan, K. M. Rabe and I. Takeuchi, *Advanced Functional Materials*, 2010, **20**, 1108-1115.
3. A. Gaur, P. Singh, N. Choudhary, D. Kumar, M. Shariq, K. Singh, N. Kaur and D. Kaur, *Physica B: Condensed Matter*, 2011, **406**, 1877-1882.
4. J. Liu, H. Deng, H. Cao, X. Zhai, J. Tao, L. Sun, P. Yang and J. Chu, *Applied surface science*, 2014, **307**, 543-547.
5. J. Liu, H. Deng, X. Zhai, H. Cao, P. Yang and J. Chu, *Journal of Materials Science: Materials in Electronics*, 2015, **26**, 2977-2981.
6. Z. Cheng, X. Wang, S. Dou, H. Kimura and K. Ozawa, *Journal*, 2008.
7. M. Yi, C. Wang, L. Li, J. Wang, Q. Shen and L. Zhang, *Applied Surface Science*, 2015, **344**, 47-51.
8. Y. Ahn and J. Y. Son, *Physica B: Condensed Matter*, 2019, **558**, 24-27.
9. Z.-Z. Jiang, Z. Guan, N. Yang, P.-H. Xiang, R.-J. Qi, R. Huang, P.-X. Yang, N. Zhong and C.-G. Duan, *Materials Characterization*, 2017, **131**, 217-223.
10. C. Daumont, S. Farokhipoor, A. Ferri, J. Wojdeł, J. Íñiguez, B. Kooi and B. Noheda, *Physical Review B*, 2010, **81**, 144115.
